# Mid-Term Audit of a National Peritoneal Surface Malignancy Program Implementation in a Low Middle Income Country: The Moroccan Experience

**DOI:** 10.3390/cancers13051088

**Published:** 2021-03-03

**Authors:** Amine Souadka, Hajar Essangri, Mohammed Anass Majbar, Amine Benkabbou, Saber Boutayeb, Laila Amrani, Abdelilah Ghannam, Brahim El Ahmadi, Zakaria Houssaïn Belkhadir, Raouf Mohsine, Abdelilah Souadka, Dominique Elias

**Affiliations:** 1 Surgical Oncology Department, National Institute of Oncology, University Mohammed V in Rabat, Rabat 10100, Morocco; essangrihajar@gmail.com (H.E.); anass.majbar@um5s.net.ma (M.A.M.); a.benkabbou@um5s.net.ma (A.B.); l.amrani@um5s.net.ma (L.A.); r.mohsine@um5s.net.ma (R.M.); abdelilah.souadka@gmail.com (A.S.); 2 Medical Oncology Department, National Institute of Oncology, University Mohammed V in Rabat, Rabat 10100, Morocco; saber.boutayeb@um5s.net.ma; 3 Intensive Care Department, National Institute of Oncology, University Mohammed V in Rabat, Rabat 10100, Morocco; abdelilah.ghannam@um5s.net.ma (A.G.); b.elahmadi@um5s.net.ma (B.E.A.); z.belkhadir@um5s.net.ma (Z.H.B.); 4 Department of Surgical Oncology, Gustave Roussy, Cancer Campus, 114 Rue Edouard Vaillant, 94805 Villejuif, France; dominique.elias@gustaveroussy.fr

**Keywords:** cytoreductive surgery, peritoneal surface malignancies, carcinomatosis, program implementation, low middle income countries, Morocco

## Abstract

**Simple Summary:**

Complete cytoreductive surgery (CRS) is the backbone of peritoneal surface malignancies (PSM) management and a major prognostic factor to better survival outcomes. Implementing a PSM program is a steep and complex process, particularly in low-middle income countries (LMIC), where limited resources are an additional challenge to overcome. In this study, we present the results of a mid-term audit of the implementation of a PSM program in Morocco. The latter was successfully and safely launched according to predicted initiation, transition and consolidation periods and allowed the significant improvement of short term surgical and oncological outcomes and completeness of cytoreduction procedures.

**Abstract:**

Implementing a multimodal management of peritoneal surface malignancies is a steep and complex process, especially as complete cytoreductive surgery (CRS) is the backbone and the major prognostic factor for hyperthermic intraperitoneal chemotherapy (HIPEC) procedures. The implementation of such a program is a challenging process, particularly in low-middle income (LMIC) countries where ressource restrictions may represent a major hurdle to HIPEC appliances acquisition. Herein is the first audit of the implementation of a national peritoneal malignancy program in a north African country. The audit process was performed according to the three implementation steps, namely initiation (“1”:2005–2008), transition (“2”:2009–2013) and consolidation (“3”:2014–2017). We included all consecutive CRS without HIPEC performed with curative intent for ovarian, gastric, colorectal and pseudomyxoma peritonei type of malignancies with an Eastern Cooperative Oncology Group (ECOG) performance Status ≤ 2. Target outcomes for incomplete cytoreduction (ICRS), serious complications ≥ 3b according to the Clavien-Dindo scoring, and early oncologic failure (EOF; disease progression within 2 years of treatment) were compared between the three phases. Independent risk factors correlated to these three outcomes were calculated using a logistic regression model.198 CRS procedures were completed with 49, 60 and 89 cases performed in the three phases, respectively. Overall, patients were comparable except for ECOG and ASA scores which were more severe in the third phase. The comparison of ICRS, serious complications and EOF rates showed a significant reduction between the three phases with (34%, 18% and 4% *p* = <0.001), (30.6%, 20% and 11.2%, *p* = 0.019) and (38.8%, 23.3% and 12.4% *p* = 0.002) respectively. Undergoing CRS in phase 3 on the other hand was a predictive factor of better short term surgical and oncological outcomes and completeness of cytoreduction, while ECOG performance status and spleno-pancreatectomy were also predictive factors of serious complications.

## 1. Introduction

Peritoneal metastases consist of malignancy dissemination within the peritoneal cavity in the form of small, white-colored tumor depositions on the inner surface of the visceral and parietal peritoneum [1,2]. Due to its dismal prognosis [3], this type of malignancy was priorly considered a terminal condition with merely palliative treatment [4]. However, cytoreductive surgery (CRS) with or without hyperthermic intraperitoneal chemotherapy (HIPEC) is nowadays increasingly considered as an alternative offering curative treatment and survival benefit [5,6].

Regardless of the type of malignancy, complete cytoreduction is a key prognostic factor that is directly linked to an increased life expectancy [7], whereas the addition of HIPEC enhances the local disease control achieved with surgery [8]. However, HIPEC requires custom made devices which can deliver chemotherapy at a steady 41–43 degrees during 30–90 min and acquiring the machine as well as its maintenance may not be affordable in low and middle income countries (LMIC) [9]. Provided that HIPEC is only complementary to CRS, the initiation of a peritoneal surface malignancy (PSM) surgical program is a crucial stepping stone to the incorporation of CRS and HIPEC in the management of peritoneal malignancies [10].

The implementation of a PSM program is a complex and challenging process with many requirements beginning with the first requisite that is a surgeon as the principal investigator that will be engaging in the coordination and initiation of the program both on an organizational and surgical level. Following, as the learning curve involves a team of surgeons, anesthesiologists, and nurses among others, the commitment to the implementation of such a program should be on an institutional level and all involved team members should be educated on the procedures. Also, good patient selection and respect of patient safety is an important component of this process, as it reflects on the post-operative morbidity and overall oncological results [11,12]. Thus far, there is no data in the literature describing the process of implementing a PSM program in a north African country. In this study, we audit the results of the implementation of a PSM program in a LMIC north African country.

## 2. Results

### 2.1. Patient Characteristics

A total of 198 CRS procedures have been completed in our institution, with 49, 60 and 89 cases performed in the three implementation phases, respectively. Of the 198 patients, 75.8% were female, with an overall mean age of 55 (range 31–78). Patients were comparable between the three periods, except for the Eastern Cooperative Oncology Group (ECOG) performance status and ASA 2 score which were more severe in the third phase. CRS was performed on PSM originating from ovarian, colorectal, pseudomyxoma peritonei and gastric cancers in 50%, 33%, 11% and 5% of the cases respectively. Preoperative chemotherapy was administered in 75% of cases. The procedure types included patients undergoing peritonectomy extending to more than four regions (46.5%), proctectomy (40%), gastrectomy (8%), spleno-pancreatectomy (3.5%), more than two bowel anastomosis (11.6%) and urology procedures (6.6%).

Further details regarding clinicopathologic characteristics, short term surgical outcomes, and their distribution according to the phases of implementation is shown in Table 1.

### 2.2. Performance of CRS

Over the three implementation phases, C0–C1 rates were 32 (65%), 49 (81.7%) and 86 (96%), respectively. Throughout the three phases, a significant decrease in ICRS (34%, 18% and 4% *p* = <0.001), severe complications (30.6%, 20% and 11.2%, *p* = 0.019) and early oncologic failure (EOF 38.8%, 23.3% and 12.4%; *p* = 0.002) rates was noted. Furthermore, despite notably elevated peritoneal carcinomatosis index (PCI), more extensive resections and higher rates of peritonectomies; the operating time, in hospital mortality and median intensive care unit (ICU) stay significantly decreased in contrast to a higher rate of CC0-CC1. Details of performance and morbidity of CRS procedures for each period is presented in Table 2.

### 2.3. Predictive Factors Incomplete Cytoreduction, Serious Complications and Early Oncologic Failure

As regards the univariate analysis, the implementation phase, sex, American Society of Anesthesiologists (ASA) score, ECOG score, primary tumor type, MDT decision, preoperative chemotherapy, PCI > 20 and spleno-pancreatectomy were statistically related to incomplete cytoreduction, G3b-5 morbidity and early oncologic failure. (Table 3) On multivariate analysis, undergoing CRS in phase 3 was a protective factor against incomplete cytoreduction (odds ratio, 0.322 [95% CI, 0.167–0.62]; *p* = 0.001), G3b-5 morbidity (odds ratio, 0.247 [95% CI, 0.114–0.534]; *p* = 0.001) and EOF (odds ratio, 0.523 [95% CI, 0.335–0.818]; *p* = 0 .004). The ECOG performance status (odds ratio, 3.776 [95% CI, 1.045–13.646]; *p* = 0.043) and spleno-pancreatectomy (odds ratio, 25.612 [95% CI, 2.314–283.435]; *p* =  0.008) were also associated with higher serious complications rates. (Table 3)

## 3. Discussion

This current study summarizes the first experience of a PSM program in Morocco, including 198 cytoreductive surgery procedures which two senior surgeons uniformly performed on peritoneal malignancies originating from ovarian, colorectal, gastric and pseudomyxoma peritonei primary tumors. This demonstrated a significant reduction in ICRS, CD3b-5 complications and EOF rates between the three phases of implementation. In addition, undergoing CRS in phase 3 proved to be a predictive factor of better short term surgical and oncological outcomes and completeness of cytoreduction, thereby indicating a progression in the learning curve and successful implementation of the PSM program in Morocco.

There are a few available guidelines on how to develop a PSM program as hospitals vary significantly in their process and timeline [12]. In our experience, we choose to progressively implement our PSM program according to three different phases each with a specific target. Accordingly, the first phase was focused on the learning process and acquiring the surgical technique to safely and adequately perform CRS. Following, the difficulty and complexity of selected cases gradually increased, allowing more patients to benefit from complete CRS and improved oncological outcomes. Similarly, surgical performance improvement was also noted. On the other hand, unlike tumor origin and PCI extent, undergoing surgery during the third implementation phase was the only independent predictive factor for CC0, thereby suggesting an improvement of radical resection through more extensive cytoreduction and increased surgical performance. This is also supported by the fact that surgery in this period is a protective factor against EOF, which reflects a more extensive CRS and the multidisciplinary team expertise. In our experience, high complications were associated with altered patient ECOG status, while undergoing a spleno-pancreatectomy was an independent risk factor of complications, probably due to the likelihood of pancreatic fistulas occurring following peritonectomies and multiple resections [13]. Unlike the surgical learning curve, demonstrating the progression of intensive care management may not be statistically possible with our data. However, during CRS and HIPEC, anaesthesiologists and intensive care physicians must manage various challenges and pathophysiological alterations in the preoperative, intraoperative and postoperative periods [14] and their team expertise has a non-negligible impact.

The management of peritoneal metastasis is one of the most challenging areas in oncology [15] with CRS viewed as the only gold standard treatment for primary and secondary PM to enable survival benefit in cases previously deemed inoperable [16]. On the other hand, the additional use of HIPEC allows the control of microscopic residual tumor tissue following successful CRS, although its indications are still disputed. In fact, with only a few randomized trials looking at the addition of HIPEC to CRS, many argue its weak supporting evidence, particularly with the protocols and outcomes differing according to the type of malignancy. [17,18] For PM originating from colorectal malignancies, the Oxaliplatin-HIPEC (PRODIGE-7) randomized study did not illustrate a significant overall survival benefit to the addition of HIPEC for patients undergoing CRS [19]. Moreover, the PROPHYLOCHIP–PRODIGE 15 trial also showed no improvement to disease-free survival in case of systematic second-look surgery plus oxaliplatin-HIPEC compared to standard surveillance for patients at high risk of developing colorectal PM [20]. As regards peritoneal carcinomatosis of gastric origin, there is scarce evidence supporting the use of HIPEC in addition to CRS [21,22]. On the contrary, despite available meta-analyses with peritoneal malignancies of ovarian origin as a topic, the benefit of HIPEC is still a subject of controversy [23]. Pseudomyxoma peritonei peritoneal carcinomatosis is the only type of tumor with consensus agreement on the indications of HIPEC and CRS [24]. However, successful cytoreductive surgery for this type of malignancy is a complex procedure with a long learning curve [5]. As can be concluded from the intersection of the aforementioned studies, the completeness of cytoreduction, surgeon experience and center expertise are the common key elements to survival benefit regardless of tumor origin. Therefore, developing proficiency in PSM surgery is the most crucial step in implementing a CRS and HIPEC specialized program [25].

The implementation of a PSM program is a complex process with a steep learning curve involving both the surgical team and the institution. Moreover, formal training for CRS does not exist in either general surgery residency, surgical oncology or gynecologic oncology fellowships [26]. In spite of that, surgeons performing this type of complex procedures must demonstrate a level of expertise above that required of the general or oncologic surgeon in order to efficiently resect and reconstruct multiple parts of the abdomen and pelvis [27,28], with experience in hepato-pancreato-biliary, advanced colorectal, and gynecologic surgery being an essential prerequisite [10]. In our experience, pelvic and ovarian surgery represented the first examples of procedures in the implementation of our PSM program (75% and 20% in the first and third phase, respectively). Advanced colorectal and esogastric surgery were also a crucial requirement as it allows the necessary technique for intestinal anastomosis. Hepatobiliary surgery, on the other hand, facilitates the exposure and mobilization of the liver for an extensive peritonectomy of the diaphragm as well as the liver hilum and fissures, especially as clearance of these structures is a requirement for complete cytoreduction. This explains the emphasis on the implementation being led by a surgeon as achieved in the first phase [11,28].

The attainment of proficiency requires a proper, long-lasting, and well-structured training of both the surgical staff and the multidisciplinary team in charge of the technical aspects of the procedure and postoperative and recovery care [26,29]. This training, alongside the consolidation of expertise in an international center, was the main target of the second phase. Ideally, the majority of teams recommend obtaining the mentorship of surgeons that are experts in PSM, however, this may only be possible on a national level, where other reference centers and skillful surgeons agree to undertake the commitment. As regards LMIC and locations with no previous reference center, this hurdle should be overcome by acquiring training and fellowships in specialized centers. Besides resorting to mentorship from centers that already succeeded in implementing such program [30], shortening the LC can be possible by developing protocols to guide the selection of patients that do not exceed the team expertise and guide postoperative complications management [31].

In a LMIC context, such as Morocco, the Implementation of a PSM program has proven to be a challenging process that may extend over a longer period due to several difficulties. Beginning the process, acquiring external mentorship may not be possible due to resource restrictions. Moreover, the length of the implementation process and its outcomes are conditioned by patient selection and choice of indications, as few patients may be available at first. Having few dedicated surgeons who are capable of performing CRS can also impede progress. Despite the foregoing, the smooth progression of the learning curve and overcoming these obstacles mostly requires long term engagement and strategic planning. This entails prevailing over the obstacle of external mentorship by pursuing a specialized fellowship and committing to the immediate initiation of the program following training.

Starting from 2018, the acquisition of HIPEC equipment enabled us to perform the first HIPEC cases, which were carefully selected in the MDT meeting and being able to ensure the LC of cytoreduction first, was a crucial step for the successful and safe addition of the HIPEC procedure. Certainly, both the CRS and HIPEC procedures could have been started at the same time from the beginning of the 3rd phase. However, we recommend for a correct start of the PSM program to avoid mixing the two different learning curves (CRS versus HIPEC). Instead, ensuring successful performance in CRS and improving its quality will leave time for the anesthesiologist and intensive care teams to learn how to manage the complexe multiple resection procedures and their postoperative recovery, before introducing the HIPEC procedure and taking the time to focus on all the specifics of chemotherapy addition and its complications.

Our Study has several limitations. The data were retrospectively collected and a low number of patients was included in our study. Also, unlike similar studies, we choose not to use the CUSUM algorithm as our aim was not to determine the required number of procedures to attain proficiency, but rather illustrate the process of implementing a program. As our cohort included peritoneal malignancies of various origins, heterogeneity may also be a limitation, the reason why instead of using the kaplan meier survival analysis to assess the oncologic outcomes, we chose a standardized variable [32]. Notwithstanding these limitations, our study documents the successful implementation of the first PSM program in north Africa that will enable patients with PSM access to curative treatment. In the long run, the continuity and growth of the PSM program activities is directly related to standardizing a specific CRS and HIPEC hands-on surgical training program, advocating health insurance coverage for PSM procedures, as well as encouraging PSM centralization by receiving referred cases from less experienced centers in the region.

## 4. Materials and Methods

This article was written according to the STROBE (Strengthening the Reporting of Observational studies in Epidemiology) directive guidelines for observational studies [33]. The study was approved by the Institutional Review Board of National Institute of Oncology in Rabat.

### 4.1. Study Overview

We conducted a retrospective audit of the implementation process of the first national PSM program in Morocco.

### 4.2. Process of Setting up a CRS Program and Training

In this cohort, the implementation process was conducted according to: (1) an initiation, (2) a transition and (3) a consolidation phase extending from 2005–2008, 2009–2013 and 2014–2017 respectively. The CRS was performed by two lead surgeons (Am S and Ab S). Initially, the initiation phase of the program was led by a senior surgical oncologist A.S. (Abdelilah Souadka), following which the transition consisted of the second surgical oncologist A.S. (Amine Souadka) enrolling in a two year sequential training in hepatobiliary (HPB), colorectal (CR) and pelvic malignancies management in a well recognized international PSM center, namely the surgical oncology department of Gustave Roussy Institute, under the mentorship of a renowned specialist (D.E.). During this period, the surgeon observed 87 CRS + HIPEC procedures and took part in perioperative management, as well as all multidisciplinary team (MDT) meetings discussing care plans for PSM cases. This period also included theoretical and surgical educational conferences, which conferred over the two years period necessary expertise for the reinforcement of the PSM program in Morocco. In the subsequent phase (consolidation), the learning curve took a more multidisciplinary and institutional aspect to bring up to speed the anesthesiology, oncology and nursing team, as well as to carry on more complex procedures and develop a care pathway. Starting from 2018, the acquisition of HIPEC equipment enabled the delivery of HIPEC to cases that are carefully selected in the MDT meeting.

### 4.3. Patient Selection

We included in our study all consecutive curative CRS without HIPEC performed in the period extending from 2005 to 2017 for ovarian, gastric, colorectal and pseudomyxoma peritonei type of malignancies. Inclusion criteria included the diagnosis of either: stage III B-IV ovarian cancer (OC), synchronous/metachronous colorectal cancer (CR), synchronous gastric cancer (GC) or symptomatic pseudomyxoma peritonei (PMP). In addition, all patients were aged between 18 and 70 years and were required to have an ECOG performance Status ≤ 2.

Preoperatively, the serum levels of tumor markers were assessed for all patients according to their type of malignancy (ACE and CA19-9 for CR, GC and PMP; CA125 for OC). Patients also underwent routine computerized tomography (CT) scan, as well as magnetic resonance imaging (MRI) in some of the cases. All cases were discussed in MDT meetings which included, alongside radiologists, medical, surgical and radiation oncologists. The imaging and files of challenging cases, for which a decision was not reached in the MDT meeting, were also discussed with DE, especially in the third phase.

### 4.4. CRS Technique

Extensive cytoreductive surgery and peritonectomy was carried out according to established procedures for the type of malignancy and extent of peritoneal carcinomatosis [34], with the latter being recorded according to the peritoneal cancer index (PCI) [35]. PCI is calculated by summing lesion size scores (0–3) in 13 regions of the abdomen with the total score ranging from 1 to 39. We categorized the PCI based on tumor origin, as well as according to the total score (< 10; 10–19; > 20).

Peritonectomies included the resection of up to five regions and were defined as follows: right and left diaphragm peritonectomy, right and left upper quadrant peritonectomy, anterior parietal peritonectomy, pelvic peritonectomy and total infragastric omentectomy. Non-excision sites were also coagulated with electrocautery.

In the case of ovarian carcinomatosis, pelvic and aortic lymphadenectomy was systematically performed in addition to CRS. If a coloproctectomy was carried out, a minimum remnant small bowel length of 2 m was needed and subsequently performing a total gastrectomy was contraindicated. For carcinomatosis synchronous to gastric cancer, partial gastrectomy was performed in addition to CRS, and Roux and Y anastomosis was performed to reconstruct the digestive tract. On the other hand, in synchronous rectal cancer, partial or total mesorectal excision was performed in addition to CRS.

All patients undergoing an unscheduled splenectomy or spleno-pancreatectomy received antibiotic therapy and vaccination during the month following surgery if no complications occurred. The completeness of cytoreduction (CC) was scored as proposed by Sugarbaker with: CC0: no residual disease; CC1: residual nodules measuring < 2.5 mm; CC2: residual nodules measuring between 2.5 mm–2.5 cm; and CC3: residual nodules > 2.5 cm [34].

### 4.5. Outcomes

For each patient, the following information was collected: age, sex, American Society of Anaesthesiologists (ASA) score, Eastern Cooperative Oncology Group (ECOG) Performance Status, type of primary tumor, MDT operative decision, preoperative chemotherapy, PCI according to tumor type and extent, as well as the type of procedure (rate of > 4 region peritonectomy, proctectomy, gastrectomy, spleno-pancreatectomy, more than two bowel anastomoses and urology procedures). We also looked at procedure related variables, namely the operating time (<6 h or >6 h), estimated blood loss (mL), in hospital mortality and median ICU stay. The main outcomes were: the completeness of cytoreduction according to CC extent, the 30-day postoperative severe complications rate (≥3b grade of the Clavien-Dindo grading system [36]) and early oncologic failure (EOF). EOF was defined as recurrence after complete cytoreduction, disease progression after incomplete cytoreduction, or death from any cause within 2 years of treatment [37].

### 4.6. Statistical Analysis

Continuous variables were presented as mean values ± standard deviations or medians with minimum/maximum, while categorical variables were expressed as frequencies and percentages. We used one-way analysis of variance (ANOVA) and Chi-Square test to analyze changes of continuous and discrete variables respectively, according to subgroups of case sequence.

The predictive risk factors for incomplete cytoreduction, G3b-5 morbidity and EOF were analysed by multivariate analysis using the logistic regression model. Clinical factors were selected as covariates when the *p* values were less than 0.1 on univariate analysis and independent factors were identified using backward stepwise selection. The odds ratios (ORs) and 95% CIs were determined for each variable and statistical significance was set at *p* < 0.05. Statistical analysis was performed using the SPSS software, version 25.0.0 (SPSS Inc., Chicago, IL, USA).

## 5. Conclusions

The learning curve of peritoneal surface malignancy surgery is a complex process requiring a specialized training and steep learning curve. We were able to successfully and safely launch a peritoneal surface malignancy program, which was carried out in accordance with the predicted diagram and following an initiation, transition and consolidation phases. The favorable surgical and oncological outcomes testify to the successful implementation of a national PSM programme in a north African country, thereby opening the perspective to complementing the programme with HIPEC and PIPAC procedures implementation, as well as pursuing designation as a national and African specialized PSM cancer center.

## Figures and Tables

**Table 1:** Patients clinical and demographic characteristics in the three implementation phases (ASA: American society of anesthesiology, ECOG: Eastern Cooperative Oncology Group, MDT: Multidisciplinary team, PCI: Peritoneal Cancer Index).

	Total (N = 198)	2005–2008 (N = 49)	2009–2013 (N = 60)	2014–2017 (N = 89)	*P*
**Age (years)**	55 (31–78)	49 (45–65)	55 (31–72)	56 (23–76)	0.7
**Sex**					
**Female**	150 (75.8)	40 (81.6)	48 (80)	62 (69)	0.19
**Male**	48 (24.2)	9 (18.4)	12 (20)	27 (31)	
**ASA score**					
1	145 (73.2)	48 (98)	47 (78)	50 (56)	0.001
2	53 (26.8)	1 (2)	13 (22)	39 (44)	
**ECOG performance Status**					
0–1	140 (70.8)	41 (83)	47 (77)	52 (58)	0.002
2	58 (29.2)	8 (17)	13 (23)	37 (41)	
**Primary tumor**					
Ovarian	100 (50)	37 (75)	45 (75)	18 (20)	0.001
CRC	66 (33)	4 (8)	10 (16,6)	52 (58)	
PMP	22 (11)	8 (16)	0	14 (15.7)	
GC	10 (5)	0	5 (8.4)	5 (5.6)	
**MDT decision**	119 (60%)	15 (30.6)	24 (40)	80 (89)	0.001
**Preoperative Chemotherapy**	147 (75)	24 (49)	49 (81)	74 (85)	0.001
**PCI per primary tumor**					
Ovarian	9 (4–18)	8 (4–10)	7 (6–12)	13 (6–18)	-
CRC	8 (5–23)	5 (3–12)	8 (5–11)	10 (8–23)	
PMP	18 (7–37)	17.5 (7–23)	0	24 (9–37)	
GC	5 (1–7)	-	5 (2-6)	5 (1–7)	
**PCI per extent**					
<10	131(66)	32 (65)	57 (95)	42 (47)	0.001
10–19	53 (26)	14 (28)	3 (5)	36 (40)	
>20	14 (8)	3 (6)	0	11 (13)	
**Procedure type**					
> 4 region Peritonectomy	92 (46.5)	6 (12.2)	6 (10)	80 (90)	0.001
Proctectomy	78 (40.)	10 (20.4)	18 (30.4)	50 (58)	0.001
Gastrectomy	16 (8)	2 (4.1)	5 (8.3)	9 (10)	0.45
Spleno-pancreatectomy	7 (3.5)	2 (4)	0	5 (5.6)	0.18
Bowel Anastomosis > 2	23 (11.6)	4 (8)	8 (13.3)	11 (12)	0.65
Urology procedures	13 (6.6)	4 (8)	1 (1.7)	8 (9.1)	0.17

**Table 2:** Performance and morbidity of CRS procedures (Early oncologic failure: Recurrence/progression or Death within the first 2years; EBL: estimated blood loss; * data unavailable).

	Total (N = 198)	2004–2008 (N = 49)	2009–2013 (N = 60)	2014–2018 (N = 89)	*P*
**Cytoreduction**					
CC0-CC1	167 (84.3)	32 (65)	49 (81.7)	86 (96)	< 0.001
CC2 (incomplete)	31 (15.7)	17 (34)	11 (18.3)	3(4)	
**Operating time < 6H**	151 (76)	30 (61.2)	43(71)	78 (87)	0.001
**Median EBL (ml)**	700	800	800	600	0.3
**Serious postoperative complication**	37 (18)	15 (30.6)	12 (20)	10 (11.2)	0.019
**In hospital mortality**	8 (4)	3(10)	3(5)	2(2,2)	0.49
**Median ICU stay**	1(1-7)	-*	3 (1-7)	1 (1-7)	0.005
**Early oncologic failure**	44 (22)	19 (38.8)	14 (23.3)	11 (12.4)	0.002

**Table 3:** Predictive factors of incomplete cytoreduction, Serious complications and early oncologic failure identified by multivariate analysis.

	Independent Risk Factors								
	Incomplete Cytoreduction			Serious Complications			EOF		
Dependent Variables	OR	95% CI	P	OR	95% CI	P	OR	95% CI	*P*
**Sex (M vs. F)**	-	-	-	1.943	0.648–5.824	0.235	-	-	-
**Primary tumor**	1.521	0.857–2.7	0.152	0.999	0.538–1.856	0.997	-	-	-
**Implementation phase (3 vs. 1/2)**	0.322	0.167–0.62	0.001	0.247	0.114–0.534	0.001	0.523	0.335–0.818	0.004
**ASA (2 vs. 0–1) (>1)**	0.884	0.245–3.193	0.851	2.388	0.503–11.342	0.274	-	-	-
**ECOG (2 vs. 0–1) (>1)**	-	-	-	3.776	1.045–13.646	0.043	-	-	-
**MDT decision**	1.461	0.58–3.68	0.421	-	-	-	-	-	-
**Preoperative CT**	0.967	0.361–2.591	0.947	0.914	0.338–3.361	0.914	0.6	0.278–1.315	0.204
**PCI Extent (>20)**	-	-	-	0.126	0.833–4.419	0.126	-	-	-
**Spleno-pancreatectomy**	-	-	-	25.612	2.314–283.435	0.008	-	-	-

## Data Availability

The data presented in this study are available on request from the corresponding author. The data are not publicly available due to privacy restrictions.
